# Glucagon-like peptide 1 level and risk of death within 90 days after intensive care unit admission: A substudy of the IVOIRE cohort

**DOI:** 10.1371/journal.pone.0323709

**Published:** 2025-05-27

**Authors:** Marine Jacquier, Annabelle Tavernier, Jean-Pierre Quenot, David Masson, Elea Ksiazek, Isabelle Fournel, Jacques Grober

**Affiliations:** 1 Service de Médecine Intensive-Réanimation, CHU Dijon, Bourgogne, France; 2 Equipe Lipness, Centre de Recherche INSERM UMR1231 et LabEx LipSTIC, Université de Bourgogne-Franche Comté, Dijon, France; 3 Centre d’Investigation Clinique, CHU Dijon, Dijon, France; 4 INSERM, CIC 1432, Module Epidémiologie Clinique, Dijon, France; 5 AgroSup, LNC UMR1231, Dijon, France; University of Illinois, UNITED STATES OF AMERICA

## Abstract

**Background:**

Elevated plasma levels of glucagon-like peptide-1 (GLP-1) have been associated with poor clinical outcome in patients with sepsis. This study investigated the association between GLP-1 levels, and survival at 90 days in a large cohort of critically ill patients.

**Methods:**

All patients aged ≥ 18 years admitted to the intensive care unit (ICU) in a large university hospital, and receiving ≥1 life support therapy for organ failure were eligible for inclusion. Plasma samples were taken within 24h of ICU admission. We measured GLP-1 using a commercial ELISA kit. Cumulative probability of death at 90 days (D90) was plotted using the Kaplan-Meier method by quartiles of GLP-1. The effect of GLP-1 quartile on D90 survival was analyzed using a Cox proportional hazards model.

**Results:**

A total of 507 patients had GLP-1 dosage; mean age 64.5 ± 14.5 years; 179 (35.3%) women. GLP-1 levels ranged from 0.03 to 129.2 (median 7.3[IQR:3.3;19.1]). Higher mean age, SOFA, SAPS II, and LPS 3HM were found in patients with higher GLP-1 quartile by univariate analysis. Overall, 229 patients (45.2%) died within 90 days. The cumulative probability of death was significantly associated with GLP-1 quartile (p log rank<0.0001). After adjustment for age, SOFA, renal replacement therapy and vasopressor treatment, a significantly increased risk was observed only for patients with the highest quartile of GLP-1 (adjusted hazard ratio 1.65 [1.06; 2.56] for 4^th^ vs 1^st^ quartile of GLP-1).

**Conclusion:**

After adjusting for demographic and clinical characteristics, only the highest quartile of GLP-1 remained independently associated with an increased risk of death at 90 days after admission to ICU.

## Introduction

Sepsis is an acute inflammatory state that is characterized by an excessive response of the body to infection, leading to activation and/or suppression of numerous systems, including the immune and metabolic pathways [1,2]. There are an estimated 48.9 million cases per year and sepsis is one of the most frequent causes of death worldwide [3]. When sepsis is complicated by septic shock, mortality was estimated to be 42% in observational study [4]. Many biomarkers have been used in recent years to assess the prognosis of patients hospitalized in the intensive care unit (ICU) for sepsis with failure of one or more vital organs. These biomarkers could be associated with mortality [5,6]. However, their utility in the diagnosis of sepsis, or in therapeutic decision-making remains uncertain [7], which limits their use in practice. In order to optimize care and support, a key clinical objective is to identify the parameters that are associated with mortality. In this regard, the digestive tract has recently been described as a distinct “organ” likely to be affected during a state of shock, but the diagnosis is often difficult based on clinical and biological parameters, and this in turn may delay timely management [8]. Intestinal dysfunction has been garnering increasing attention and shock conditions seem to be associated with mesenteric ischemia [9]. Indeed, impaired gut barrier function (“leaky gut”) might enable toxic molecules such as lipopolysaccharide (LPS) to pass into the systemic circulation, promoting systemic inflammation, thus organ failure and increased mortality [10–12]. Lesions at the level of the enterocytes have also been suggested to result from non-occlusive mesenteric ischemia, due to an abrupt mismatch between supply and demand of oxygen and nutrients in the splanchnic zone [11,12]. Piton et al investigated a cohort of ICU patients with shock and observed a correlation between low plasma levels of intestinal fatty-acid binding protein (iFABP) or citrulline, and mortality at 28 days [13]. More recently, a hormone secreted by intestinal cells, namely Glucagon-Like Peptide 1 (GLP-1), has been proposed as a marker of gut barrier dysfunction [14]. Indeed, it was shown by our group to be associated with LPS translocation, inflammation and adverse outcome, in patients undergoing cardiac surgery [14].

Although first described as a glucoregulatory incretin hormone [10], GLP-1 also has potent anti-inflammatory properties [15]. Plasma GLP-1 levels are increased by lipopolysaccharide (LPS) [16] and GLP-1 is rapidly secreted when the gut barrier is impaired [17]. Elevated plasma levels of GLP-1 are associated with poor clinical outcome in patients with sepsis [18]. GLP-1 could thus be a potential early signal of intestinal ischemia, and consequently, may be associated with mortality.

This observational study aimed to explore the association between GLP-1 levels and survival at 90 days in a large cohort of critically ill patients.

## Patients and methods

### Study population

**Fig 1:**
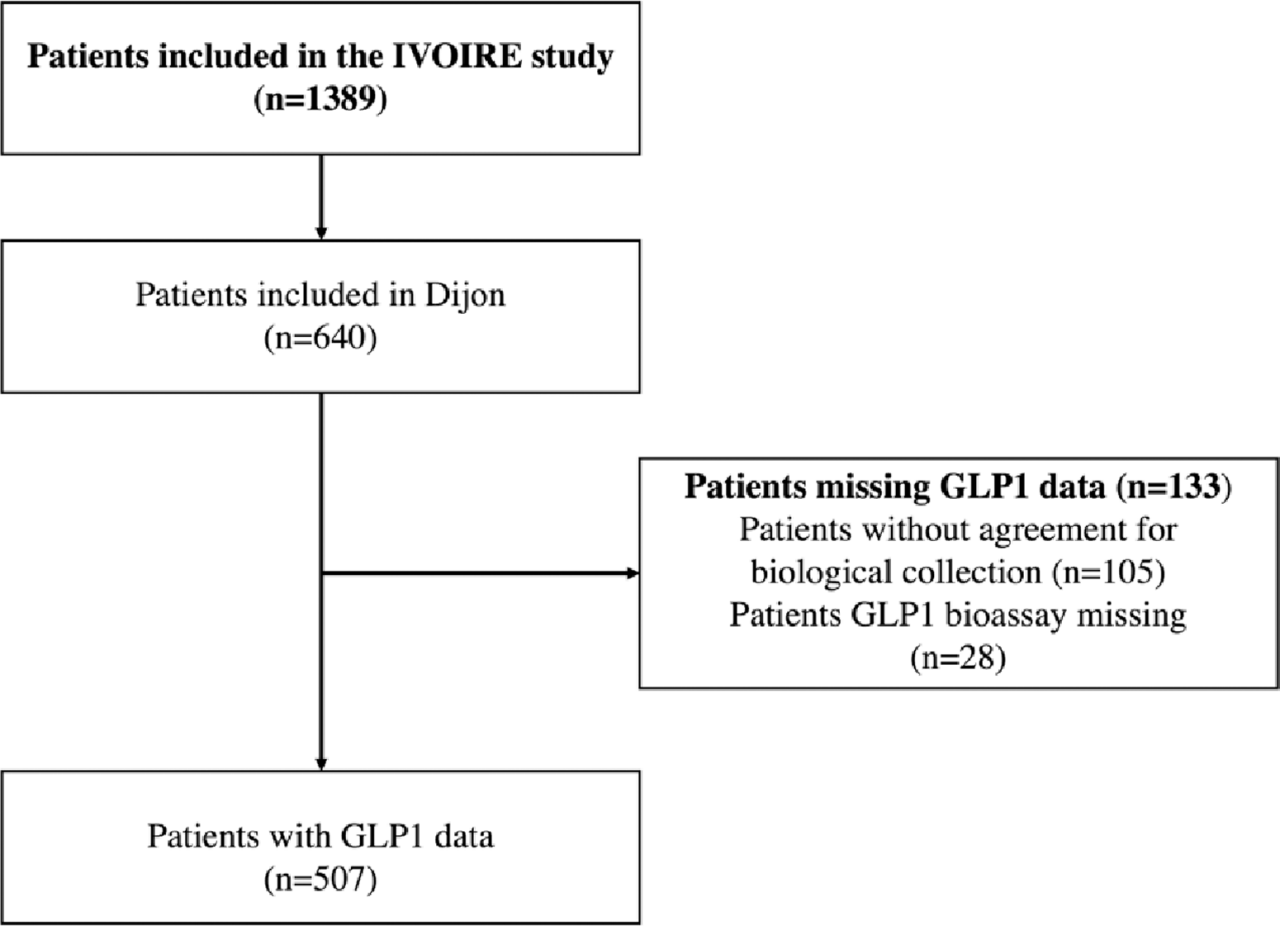
Flowchart of patients from IVOIRE study included in the analysis of GLP-1 levels.

This study used data from the French multicenter IVOIRE cohort study (Fig 1), an observational study designed to assess the impact of socio-economic deprivation on the severity of illness at ICU admission, and on mortality [19]. Briefly, all patients aged ≥ 18 years admitted to the ICU in 8 French participating centers and receiving at least one life support therapy for organ failure were eligible for inclusion.

For the present substudy, only critically ill patients enrolled in the ICU of Dijon university hospital were included, due to the receipt of specific funding for collection of biological samples in this center. This was the coordinating centre of the study, and the staffing and technical plateau enabled the blood sampling and analysis. Patients were included between 08/06/2013 and 26/09/2017. The specific schedule for this substudy has previously been described [20]. Briefly, patients (or their next of kin) were informed and signed an additional, specific consent form authorizing the conservation and utilization of biological samples. Plasma samples were collected on the day of inclusion (<24 hours since ICU admission, Day 1). The samples were processed following a strict protocol, maximum 1 hour after sampling, with protocolized centrifugation and immediate freezing at -80°C, then transfer for storage at the biobank facility of Dijon University Hospital (Centre de Ressources Biologiques Ferdinand Cabanne).

The main IVOIRE study received approval from the institutional research committee (Comité de Protection des Personnes (CPP)), on 21 February 2013, under the number 2013-A00095-40 (full study title in French: “Influence de la vulnérabilité socio-économique sur la gravité initiale et le pronostic des patients admis en réanimation. Etude de cohorte, prospective, multicentrique”). All study procedures were performed in accordance with the ethical standards of the institutional research committee, national legislation in relation to biomedical research involving human subjects, and the Helsinki Declaration of 1975 and its subsequent amendments.

### Data collection

The main variable of interest in the present analysis was GLP-1 level. Other data collected included demographic characteristics, body mass index (BMI), level of education, social deprivation, comorbidities (Charlson index, diabetes), severity at baseline (Simplified Acute Physiological Score (SAPS II) and Sequential Organ Failure Assessment (SOFA) scores at ICU admission, type of life support therapy (mechanical ventilation, vasopressors and/or inotropic agent, renal replacement therapy (RRT), high flow nasal cannula), length of ICU stay, documented infection within 24 hours after ICU admission and 3HM (3-hydroxymyristate) levels. The main outcome was survival at D90 (90 days after ICU admission).

### GLP-1 analysis

Total GLP-1 concentrations were determined by commercially available ELISA kits (EZGLP1T-36K, Millipore) in accordance with the manufacturer’s protocol. GLP-1 was assessed on plasma. Intra- and inter-sample coefficients of variation were 3.2% to 6.7% and 5.4% to 8.7% respectively. A control set was measured for calibration. This technique has been widely used and validated in previous studies [17].

### LPS

Levels of 3-HM (LPS) plasma concentrations were determined using a mass spectrometry (LC-MS/MS) patented method (EndoQuant) for detection of 3-OH fatty acids (C10, C12, C14, C16 and C18), which are molecules bound to the lipid A motif of LPS. Detailed methods have previously been described elsewhere by our team [21]. This method seems to present major advantages compared to the classical method of dosing LAL (Lysosomal Acid Lipase) [20].

### Statistical analysis

Quantitative variables are presented as mean±standard deviation (SD) or median (Inter-Quartiles, quartile (Q1), Q3) as appropriate. Qualitative variables are presented as number and percentage.

As the distribution of GLP-1 level was not normal, GLP-1 levels were categorized into quartiles. Characteristics of patients according to GLP-1 quartiles were compared using Chi2, ANOVA or the Kruskal Wallis test, as appropriate.

Cumulative probability of death at 90 days (D90), defined as the time from ICU admission until death from any cause, was plotted using the Kaplan-Meier method according to quartiles of GLP-1, and compared using the log-rank test. The effect of GLP-1 quartile on survival at D90 was analyzed using a Cox proportional hazards model. A crude model was constructed first, followed by a multivariate model. Candidate variables for multivariate analysis were age, sex, SOFA and SAPSII scores at admission, infection within 24h following ICU admission, vasopressors during ICU stay, RRT during ICU stay, Charlson comorbidity index, 3-HM, ICU length of stay and BMI. Variables associated with survival at D90 in bivariate analysis with a p-value <0.20 were included as covariates in the multivariable Cox model. A backward selection procedure (p-value <0.10 to stay in the model) was applied to obtain the final model, and the GLP-1 level was forced. The assumption of proportionality was checked graphically using cumulative sums of martingale residuals. Collinearity between adjustment variables was verified by calculating the variance inflation factor (VIF); all values were < 5, indicating absence of multicollinearity. All analyses were performed using SAS version 9.4 (SAS Institute Inc., Cary, NC, USA) with a significance threshold set at 0.05.

## Results

Among the 640 patients included in the IVOIRE cohort by Dijon University Hospital, 507 (79.2%) had GLP-1 dosage (Fig 1). Compared to patients without GLP-1 dosage, patients with GLP-1 dosage were more often males (54% vs 65%, p = 0.02) but there were no significant differences in terms of other socio-demographic variables (age, level of education, social deprivation) or clinical variables (BMI, severity at baseline, smoking) (S1 Table).

The 507 patients (of whom 179 (35.3%) women) with GLP-1 dosage were aged 64.5 ± 14.5 years on average. GLP-1 levels ranged from 0.03 to 129.2 ng/mL, with a median value of 7.3 ng/mL [IQR:3.3;19.1], and 3HM levels ranged from 8.7 to 547.7, with a mean value of 123.4 ± 72.2.

The characteristics of the critically ill patients according to GLP-1 distribution are presented in Table 1. Higher mean age, SOFA, SAPS II, and LPS 3HM were found in patients with higher GLP-1 levels. There were also significantly more patients with infection within 24h after ICU admission, patients treated with vasopressors or RRT among those with higher GLP-1 levels. The proportion of women was significantly lower at higher levels of GLP-1.

**Table 1:** Characteristics of critically ill patients and controls, in the population according to quartiles of GLP-1.

	GLP-1 levels (ng/mL)				p-value*
	1^st^ quartile < 3.25 n = 126 N (%) Mean ± SD Median [Q1; Q3]	2^nd^ quartile [3.25–7.31] n = 127 N (%) Mean ± SD Median [Q1; Q3]	3^rd^quartile [7.32–19.01] n = 127 N (%) Mean ± SD Median [Q1; Q3]	4^th^ quartile ≥ 19.09 N = 127 N (%) Mean ± SD Median [Q1; Q3]	
Women	59 (46.8)	49(38.6)	35 (27.6)	36 (28.4)	**0.003** ^**1**^
Age	62.9 ± 15.5	68.2 ± 15.0	68.6 ± 13.7	70.2 ± 12.8	**<0.001²**
BMI	28.3 ± 8.6	27.9 ± 8.3	26.5 ± 5.9	28.6 ± 6.8	0.130²
Level of education					0.209
No diploma	40 (32.8)	27 (22.0)	25 (20.5)	31 (25.2)	
Secondary level	32 (26.2)	38 (30.9)	42 (34.4)	42 (34.2)	
Vocational diploma	26 (21.3)	39 (31.7)	37 (30.3)	26 (21.1)	
High school diploma and higher	24 (19.7)	19 (15.5)	18 (14.8)	24 (19.5)	
Smoking status					0.162
Non smokers	46 (38.0)	45 (36.0)	48 (38.1)	52 (41.3)	
Current smokers	46 (38.0)	36 (28.8)	42 (33.3)	29 (23.0)	
Former smokers	29 (24.0)	44 (35.2)	36 (28.6)	45 (35.7)	
< 3 ADLs (Katz)	9 (7.3)	8 (6.5)	5 (4.0)	5 (4.1)	0.569^1^
SOFA	7.1 ± 3.5	8.7 ± 3.4	9.8 ± 3.4	11.9 ± 3.6	**<0.001²**
SAPS II	47.9 ± 16.6	51.6 ± 18.5	55.6 ± 14.9	65.8 ± 20.0	**<0.001²**
Charlson index ≥ 3	51 (40.5)	57 (44.9)	62 (48.8)	67 (52.8)	0.238^1^
Diabetes	33 (26.2)	44 (34.7)	31 (24.4)	41 (32.3)	0.227^1^
Infection within 24h of ICU admission	72 (51.1)	97 (78.2)	105 (83.3)	110 (86.6)	**<0.001** ^**1**^
Lung	47 (65.3)	62 (63.9)	72 (68.6)	63 (57.8)	0.424^1^
Digestive	7 (9.7)	4 (4.1)	7 (6.7)	11 (10.1)	0.357^1^
Urinary tract	37 (51.4)	55 (56.7)	49 (46.7)	56 (51.4)	0.566^1^
Other	16 (22.2)	30 (30.9)	31 (29.5)	33 (30.3)	0.598^1^
Treatments					
Invasive ventilation	104 (82.5)	95 (74.8)	102 (80.3)	107 (84.3)	0.251^1^
NIV	35 (27.8)	27 (21.3)	23 (18.1)	24 (18.9)	0.229^1^
Vasopressors	65 (51.6)	90 (70.9)	98 (77.2)	116 (91.3)	**<0.001** ^**1**^
RRT	15 (11.9)	20 (15.8)	39 (30.7)	58 (45.7)	**<0.001** ^**1**^
Length of ICU stay (days	4 [2; 10]	5 [2; 9]	5 [3; 9]	6 [3; 13]	0.063^3^
3HM levels	112.4 ± 74.3	110.7 ± 53.5	128.3 ± 80.0	142.2 ± 74.3	**0.001²**

*p-value for comparison between patients’ characteristics and GLP-1 levels.

p-value from ^1^chi-2 test, ^2^Student test and ^3^ Kruskal-Wallis test

NIV: non-invasive ventilation; RRT: renal replacement therapy; SAPS: simplified acute physiology score; SOFA: sequential organ failure assessment; 3 HM: 3-hydroxy myristate.

Missing data: level of education (n = 17), ADL Katz (n = 12), BMI (n = 3), infection within 24h after intensive care unit admission (n = 4) Lung infection (n = 1), digestive infection (n = 1), urinary tract infection (n = 1), other infection site (n = 1)

**Fig 2:**
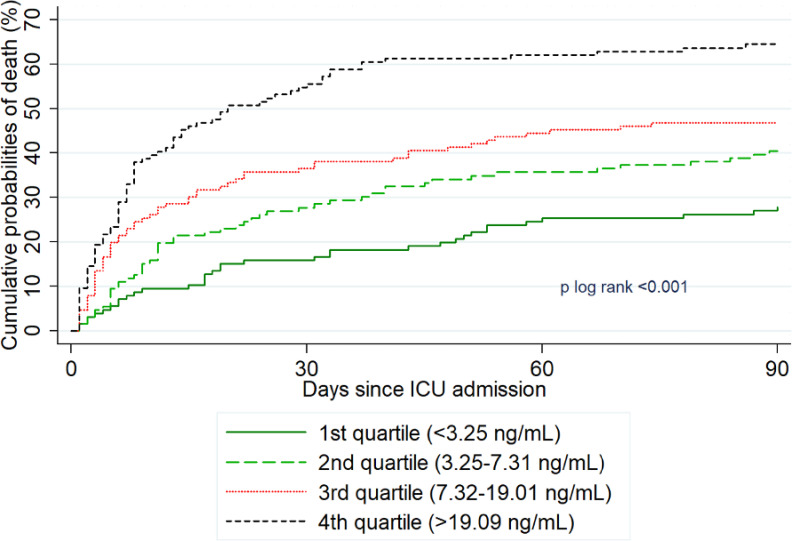
Cumulative probabilities of death and risk of death at D90 according to GLP-1 quartile.

Overall, 229 patients (45.2%) died within 90 days following admission. The cumulative probability of death was significantly associated with GLP-1 level (p log rank<0.0001, Fig 2; Table 2), with a cumulative probability of death ranging from 27.8% for the lowest GLP-1 quartile to 64.5% for the highest quartile. After adjustment for age, SOFA, vasopressor treatment and RRT, only a trend toward a higher risk of death with GLP-1 levels was found (p = 0.099), with significantly increased risk observed only for the patients in the highest quartile compared to the lowest quartile (adjusted hazard ratio 1.65[95%CI:1.06–2.56], Table 2).

**Table 2:** Effect of GLP-1 levels on risk of death within 90 days after ICU admission.

	Cumulative probability of death at D90 (%)	Crude HR [95% CI] (n = 507)	p- value	Adjusted HR* [95% CI] (n = 503)	p- value
GLP-1 levels			**<0.001**		**0.099**
1^st^ quartile (GLP-1 < 3.25 ng/mL)	27.8%	Reference		Reference	
2^nd^ quartile (3.25–7.31 ng/mL)	40.4%	1.58 [1.03; 2.43]		1.14 [0.73; 1.77]	
3^rd^ quartile (7.32–19.01 ng/ml)	46.8%	2.07 [1.36; 3.14]		1.30 [0.84; 2.01]	
4^th^ quartile GLP-1 > 19.09 ng/mL	64.5%	3.50 [2.36; 5.20]		1.65 [1.06; 2.56]	

* Adjusted for age, RRT during ICU stay, vasopressive treatment and SOFA. HR, hazard ratio; CI, confidence interval.

## Discussion

This study shows a trend toward a higher risk of death with GLP-1 levels. After adjustment for demographic and clinical characteristics, only the highest GLP-1 level (assessed in quartiles) remained significantly associated with the risk of death at 90 days among critically ill patients admitted to ICU. To the best of our knowledge, this is the largest cohort of critically ill patients to date to investigate the potential association between GLP-1 and survival in ICU patients.

Our findings are promising, and build upon a growing body of evidence linking elevated GLP-1 levels to adverse outcomes in critically ill patients. Several studies have demonstrated this association in various contexts, including myocardial infarction complicated by cardiogenic shock [22] and sepsis [18]. Kahles et al. showed that high GLP-1 levels at admission in patients with myocardial infarction and cardiogenic shock were a strong predictor of one-year mortality [22]. This line of evidence suggests that GLP-1 elevation may play different roles in different organs during sepsis, including cardiac stress and renal dysfunction. Similarly, Brakenridge et al. found that persistently elevated GLP-1 in critically ill surgical patients with sepsis was associated with chronic critical illness and poor long-term outcomes [18]. A recent review also discussed the role of GLP-1 and its receptor agonists in sepsis, indicating that patients with sepsis have significantly higher GLP-1 levels, which are associated with worsening of disease and death [23]. The review also highlighted that GLP-1 receptor agonists might help control blood sugar, protect organs, and modulate inflammatory responses [23].

GLP-1 plays a key role in glucose homeostasis, specifically by stimulating secretion of insulin, suppressing secretion of glucagon, inhibiting gastric emptying and reducing appetite. Disruption of glucose homeostasis due to a pro-inflammatory environment and the response to injury is frequent in critically ill patients in the ICU, especially in those with sepsis [24]. Deane et al reported that the administration of a synthetic analogue of human GLP-1, exenatide, was associated with a reduction in hypoglycaemic episodes and postprandial glycaemic excursions in ambulant type 2 diabetic patients [25]. Indeed, there is a link between the inability to control hyperglycemia, and an increase in nosocomial infections and mortality [26–28]. In our study, we did not observe any hyperglycemic episodes, but all patients in our study were admitted to ICU and had strict glycemic control according to current recommendations [29,30]. We have information about the presence of diabetes in our patients (and this was taken into account in the analysis), but not the full details of stop and start dates for insulin or other types of treatment, which could be a limitation of our study. In diabetic patients and indeed all patients with insulin resistance, the presence of both hyperglycemia and genetically determined GLP-1 resistance can potentiate the impairment of GLP1-induced insulin secretion [31]. Only 26 patients were receiving GLP-1 agonists, with or without another treatment in the whole population, limiting a potential effect. Nevertheless, we cannot exclude the possibility that residual confounding from unmeasured risk factors may account for the observed associations. The GLP-1 levels could be changed in these patients, and this situation should be taken into account in future studies.

Brakenridge et al previously reported in post-operative patients who developed sepsis that the GLP-1 level in the 24 hours after onset of sepsis was a predictor of early death and persisting organ failure [18]. These authors also highlighted that persistent elevation of GLP-1 levels in survivors was a marker of an unresolved catabolic state associated with loss of muscle mass and unfavourable short-term prognosis in terms of functional capacity and death [18]. Lebherz et al. also reported the predictive value of GLP-1 levels in ICU patients with chronic renal failure receiving hemodialysis, compared to a control group of patients with neither inflammation nor renal dysfunction [32]. In our study, we did not explore the correlation between acute renal failure and GLP-1 levels.

It has been shown that GLP-1 increases rapidly in response to inflammation [33]. In our study, we did not measure circulating levels of cytokines, but indirectly, we can hypothesize that patients with the highest SOFA scores were also those with the greatest degree of inflammation, and these were also the patients with the highest GLP-1 levels. Piton et al underline an association between vasopressors and gut suffering [13]. There is undoubtedly a strong link between intestinal dysfunction, reflected by circulating GLP-1, and the inflammation prompted by passage of LPS from the gut into the blood circulation. However, this remains a hypothesis that we cannot substantiate with our study, since we did not perform additional examinations to diagnose mesenteric ischemia, for example. Until recently, direct mass quantitation of LPS using mass spectrometry was not adapted to complex matrices such as human plasma [33]. Our research unit developed a new method for LPS quantitation using 3-hydroxy myristate (3HM), a specific component of the lipid A (the lipid moiety of LPS) [34] detected using high performance liquid chromatography coupled with tandem mass spectrometry (LC-MS/MS) [21]. We observed that 3HM was significantly higher in patients with the highest GLP-1 levels. After remodelling of gut integrity, it was also reported that plasma GLP-1 levels are rapidly increased in mice, even before a perceptible change in LPS plasma levels (secreted by intestinal cells). Moreover GLP-1 has been shown to be correlated with levels of pro-inflammatory cytokines after cardiac surgery, such as growth differentiation factor 15 (GDF-15), a cytokine induced by the stress linked to transforming growth factor beta [14,32].

Gut barrier dysfunction is one of the potential mechanisms linking elevated GLP-1 to poor outcomes involves. In critical illness, particularly sepsis, compromise of the gut barrier leading to translocation of LPS and other bacterial products into the systemic circulation may be reflected by elevated GLP-1. Indeed, it has been suggested that GLP-1 plays a role in gut-immune interactions by modulating innate immune responses in a number of inflammatory settings [35]. In sepsis, the increased LPS translocation contributes to systemic inflammation, potentially exacerbating the patient’s condition.

Clearly, the role of GLP-1 in critical illness presents a paradox. While it possesses known anti-inflammatory properties, elevated levels are consistently associated with worse outcomes. This could be a compensatory mechanism, or it could indicate that the normal regulatory functions of GLP-1 are impaired in severe sepsis, or reflect the severity of the underlying disease process. Further investigation is needed to disentangle these possibilities and determine whether GLP-1 elevation is a contributor to pathogenesis, a protective mechanism or a marker of disease severity. Our findings suggest that it is likely a complex interplay of all three. While GLP-1 may initially be released as part of a protective response, its sustained elevation in the context of severe critical illness may indicate a failure of this response and contribute to the pathogenesis of organ dysfunction. It would be useful to differentiate between treatments like GLP-1 analogues, which reduce inflammation partly by reducing the secretion of pro-inflammatory cytokines (e.g., IL-6 or TNF) [36] on the one hand; and endogenous secretion of GLP-1 on the other hand, which is activated in sepsis via activation of the innate immune system and associated with organ dysfunction [36,37]. Furthermore, anti-inflammatory treatments are currently debated in sepsis given the expanding understanding of septic immunoparalysis. A number of anti-inflammatory therapies have failed to show any benefit in this setting and it is possibly via their effect in reducing oxidative stress that they show the most promising activity [38]. Understanding these mechanisms will be vital for integrating GLP-1 into clinical decision-making and therapeutic strategies.

Our study remains an exploratory study, but GLP-1 analysis nevertheless holds promise for clinical practice, because of its ease of implementation. As an early signal or by contributing to an early diagnosis of gut impairment, or as an additional argument for sepsis, GLP-1 could be a useful addition to the diagnostic armamentarium, and shows promise as a prognostic biomarker in critically ill patients. Its potential utility lies in its ability to signal a higher risk of mortality, making it a valuable tool for early intervention and risk stratification. Future research should aim to explore the prognostic value of GLP-1 further, especially in multicenter studies with diverse patient populations and comprehensive biomarker analysis. For example, future prospective studies should investigate the relationship between GLP-1 levels and the diagnosis of mesenteric ischemic, ideally coupled with CT scan data and lactate levels, and interpret the results according to different glucose levels. It would also be informative to study GLP-1 levels in relation to other markers of pro-inflammatory activity (IL-6 or IL-8 for example), or markers of septic immunoparalysis such as monocytic deactivation (HLA-DR), in order to obtain a more personalized profile for each patient.

## Limitations

This study has some limitations. Firstly, this study was designed to explore the association between GLP-1 levels and survival, and consequently did not enable us to assess the performance of GLP-1 as an early predictor of death. In addition, it is a single-centre study, which limits its external validity. Indeed, blood samples were not collected in other centers, and therefore, the external validity, required in prediction studies, is limited. Moreover, the characteristics of patients differed between our centre and the patients from other centres in the IVOIRE trial. Notably, patients in our centre had a more severe profile, and more often presented severe sepsis or septic shock. Nevertheless, we included a large spectrum of patients likely to be representative of the population of patients usually admitted to ICU, and it is precisely in these patients that it is useful to know which parameters are associated with a significantly increased risk of mortality. Secondly, this is a *post hoc* analysis of an observational study. Third, specific data on renal function, exact control of glycemia, and specific antidiabetic treatments were not collected. Although SOFA score, which includes renal function, and RRT were both taken into account in the analysis, we cannot exclude the possibility that residual confounding from unmeasured risk factors may account for the observed associations. Finally, the biological samples were drawn at the time of inclusion, with a single-timepoint, cross-sectional assessment of GLP-1 level. This reliance on a single measurement of GLP-1 at ICU admission may not fully capture its prognostic potential. Although all patients were included in the study within 24 hours of ICU admission, there may be some variation between the onset of septicaemia (for patients with sepsis) and the time of the blood draw, which may not be fully representative of the initial biological state. The course of GLP-1 levels over time might have been more informative. Future studies should consider serial measurements of GLP-1 levels (e.g., daily during ICU stay), to provide a more comprehensive understanding of the dynamic changes in GLP-1 levels, and to determine whether sustained or transient elevations offer differential prognostic value. Specifically, investigation of a potential correlation with lactate levels and signs of mesenteric ischemia on abdominal CT are warranted.

## Conclusion

A trend toward a higher risk of death with rising GLP-1 levels was observed. The highest quartile of GLP-1 concentrations was independently associated with an increased risk of death at 90 days after admission to ICU, compared to the lowest quartile. Future studies are warranted to validate our findings, and further elucidate the exact role of GLP-1 in critically ill patients, especially those with sepsis, taking into account possible confounders such as renal dysfunction. Within the limitations of this study, GLP-1 has potential clinical relevance as a biomarker for guiding therapeutic strategies in critically ill patients.

## Supporting information

S1 Table Comparison of the socio-demographic and clinical characteristics between patients included in Dijon University Hospital and those included in other centres from the IVOIRE study. (DOCX)
