# Supplementary material for: Glucagon-like peptide 1 level and risk of death within 90 days after intensive care unit admission: A substudy of the IVOIRE cohort
Source: PLoS One. 2025 May 27;20(5):e0323709. doi: 10.1371/journal.pone.0323709 (PMC12111264; doi:10.1371/journal.pone.0323709)
Supplement: S1 Table — (DOCX) [file pone.0323709.s001.docx]

**Supplementary Table S1 : Comparison of the socio-demographic and clinical characteristics between patients included in Dijon University Hospital and those included in other centres from the IVOIRE study**

| **Variable** | **Overall** | **Dijon** | **Other centres** | **p-value** |
| --- | --- | --- | --- | --- |
| **Age**, years | 66.26±15.05 | 67.66±14.17 | 65.07±66.84 | 0.001 |
| **Sex** |  |  |  | 0.83 |
| Male | 864 (62.20) | 400 (62.50) | 464 (61.95) |  |
| Female | 525 (37.80) | 240 (37.50) | 285 (38.05) |  |
| **SAPSII score** | 51.40±18.62 | 54.80±19.12 | 48.50±17.68 | <0.001 |
| **SOFA score** | 7.95±4.06 | 9.03±4 | 7.02±3.89 | <0.001 |
| **Alcohol consumption** |  |  |  | <0.001 |
| <2 units per day | 906 (68.12) | 394 (63.14) | 512 (72.52) |  |
| 2 or more units/day | 424 (31.88) | 230 (36.86) | 194 (27.48) |  |
| **Smoking status** |  |  |  | <0.001 |
| Non smoker | 509 (37.10) | 251 (39.97) | 258 (34.68) |  |
| Current smoker | 379 (27.62) | 191 (30.41) | 188 (25.27) |  |
| Former smoker | 484 (35.28) | 186 (29.62) | 298 (40.05) |  |
| **Highest level of education** |  |  |  | <0.001 |
| No diploma | 259 (19.56) | 146 (23.59) | 113 (16.03) |  |
| Primary school | 377 (28.47) | 207 (33.44) | 170 (24.11) |  |
| Vocational diploma | 380 (28.70) | 161 (26.01) | 219 (31.06) |  |
| High school diploma or higher | 308 (23.26) | 105 (16.96) | 203 (28.79) |  |
| **Charlson score** |  |  |  | 0.07 |
| 0 to 2 | 798 (57.45) | 351 (54.84) | 447 (59.68) |  |
| >= 3 | 591 (42.55) | 289 (45.16) | 302 (40.32) |  |
| **SIRS** |  |  |  | <0.001 |
| Yes | 1053 (75.81) | 513 (80.16) | 540 (72.10) |  |
| No | 336 (24.19) | 127 (19.84) | 209 (27.90) |  |
| **Severe sepsis or septic shock <24h after admission** |  |  |  | <0.001 |
| Yes | 766 (55.15) | 456 (71.25) | 310 (41.39) |  |
| No | 623 (44.85) | 184 (28.75) | 439 (58.61) |  |
| **Length of stay in ICU,** days | **9.88±13.85** | 8.56±13.68 | 11.01±13.90 | <0.001 |
| **Death at 90 days** |  |  |  | <0.001 |
| Yes | 439 (31.61) | 282 (44.06) | 157 (20.96) |  |
| No | 950 (68.39) | 358 (55.94) | 592 (79.04) |  |

SAPSII, Simplified Acute Physiology Score; SOFA, Sequential Organ Failure Assessment; SIRS, Systemic inflammatory response syndrome; ICU, intensive care unit.
